# Advertising

**Published:** 1875-09

**Authors:** 


### Editorial.

THE OTHER SIDE OF THE MATTER of proprietary advertising is put in a pointed way by a recent communication from another of our subscribers:

"I have just read your editorial, 'An Honest Confession is Good for the Soul,' and I trust that the censor's anxieties and apprehensions, lest the 'profession be circumvented,' are somewhat quieted. The subscriber would not only make the editors and publishers the guardians of the perplexed, the unwary and the credulous, but he seems unwilling that 'the proprietary scoundrels' should assist in furnishing him with a most excellent medical journal. He submits to you, 'should such things be?' If not, then let the subscribers furnish the remedy—that is, increase their subscription fees so as to prevent the necessity and render the journal independent of all such outside aid. Don't you concede too much to the fastidious complaints of your subscriber? Isn't there a palpable distinction between the journal proper and the advertising sheets that envelop it? 'A proper degree of circumspection' should certainly be incumbent upon the readers of advertisements, and they alone should be held responsible for the extent to which they may be influenced by the blarney, the trappings and the paraphernalia."

